# Testing the theory of planned behavior in predicting evacuation intentions during storms

**DOI:** 10.1371/journal.pone.0361994

**Published:** 2026-11-02

**Authors:** Amer Hamad Issa Abukhalaf, Abdallah Y. Naser

**Affiliations:** 1 Nieri Department of Construction and Real Estate Development, Clemson University, Clemson, South Carolina, United States of America; 2 Department of Applied Pharmaceutical Sciences and Clinical Pharmacy, Isra University, Amman, Jordan; Shanghai University, CHINA

## Abstract

The Theory of Planned Behavior (TPB) has been widely applied to predict human behavior across diverse domains; however, its application in disaster contexts remains limited. This study addresses this gap by examining the utility of TPB in predicting intentions to prepare evacuation plans in hurricane-prone areas. Using a quantitative approach, an online survey was administered to adults residing in Florida, yielding 816 valid responses. Attitude toward evacuation planning, subjective norms, and perceived behavioral control were measured using validated Likert-scale items. Data were analyzed using Spearman’s correlation and binary logistic regression. Results indicate that all TPB constructs were significantly associated with evacuation planning intentions. Subjective norms emerged as the strongest predictor, highlighting the critical role of social influence in preparedness decisions. Attitude toward evacuation planning and perceived behavioral control also demonstrated significant positive effects, suggesting that favorable evaluations of evacuation planning and confidence in one’s ability to prepare increase preparedness intentions. The logistic regression model showed strong predictive power (Nagelkerke R^2^ = 0.62). These findings support the applicability of TPB in disaster preparedness research and underscore the importance of integrating social norms, perceived behavioral control, and attitude-based interventions into emergency management strategies.

## 1. Introduction

The Theory of Planned Behavior (TPB) has been widely used as a robust and flexible framework for explaining and predicting human behavior across a broad range of domains. Originally developed by Dr. Ajzen, the theory posits that behavior is primarily driven by a behavioral intention, which is shaped by several behavioral constructs [1]. Over the past several decades, TPB has demonstrated strong explanatory and predictive power in areas such as public health, transportation, environmental behavior, consumer decision-making, and safety compliance [1–3]. Researchers have successfully applied the theory to behaviors including physical activity, vaccination uptake, recycling, travel mode choice, and adherence to safety regulations, often reporting good predictive validity [2–4]. Its success stems from its ability to integrate individual beliefs, social influences, and perceived constraints into a single coherent framework, making it one of the most influential and frequently used behavioral theories in the social sciences. Despite its extensive application and empirical support, the use of TPB to predict and explain behavior during disasters remains limited [5,6]. And this is the research gap that this study is aiming to fill.

Storms continue to pose one of the most significant natural hazards worldwide, causing substantial loss of life, injuries, economic disruption, and damage to critical infrastructure. In recent decades, increasing coastal development, population growth in hazard-prone regions, and the growing intensity of extreme weather events have amplified the consequences of hurricanes and tropical storms [5,7]. As more communities become exposed to these hazards, improving disaster preparedness has become a central objective of emergency management agencies. Among the various protective actions available, timely evacuation remains one of the most effective strategies for reducing casualties and protecting public safety. Consequently, understanding the factors that influence evacuation preparedness and planning has become an increasingly important area of disaster research [6,8].

Despite widespread public education campaigns and advances in weather forecasting, many individuals fail to prepare for evacuation or choose not to evacuate even when official warnings are issued. Such decisions are rarely based solely on hazard information; rather, they are shaped by a complex interaction of psychological, social, and contextual factors [5,6]. Individuals often weigh perceived risks, previous disaster experiences, expectations of family and friends, and their confidence in their ability to evacuate before deciding whether to prepare. These behavioral processes create significant challenges for emergency managers, highlighting the need for theoretical frameworks capable of explaining why individuals intend to prepare for evacuation and identifying opportunities to improve preparedness through evidence-based interventions [7,8]. Disaster situations differ fundamentally from routine decision-making contexts due to heightened uncertainty, emotional stress, time pressure, and rapidly evolving risk conditions. These characteristics may alter how individuals evaluate risks, perceive social expectations, and assess their ability to act [7].

Against this background, the present study applies the TPB to examine intentions to prepare evacuation plans among adults living in hurricane-prone areas. Specifically, the study evaluates whether attitude toward evacuation planning, subjective norms, and perceived behavioral control are associated with individuals’ intention to prepare an evacuation plan during the next hurricane season. By examining these relationships in the context of hurricane preparedness, this study extends the application of the TPB to disaster-related decision-making and provides evidence that may inform future emergency-management communication and preparedness interventions. The next section reviews the theoretical and empirical literature supporting these relationships.

## 2. Literature review

The TPB serves as a framework for explaining and predicting numerous human behaviors. The purpose of introducing the TPB was in fact to improve the predictive power of the original theory; Theory of Reasoned Action, which Dr. Ajzen introduced in 1980 [7]. According to both of these theories, if a person evaluates a certain behavior as good/positive, and if he/she believes that important/close people in their life want them to perform that behavior, the intention to perform the behaviors becomes even greater and they will be more likely to do them [1,2]. Since behavioral intention doesn’t necessarily lead to actual behavior, Dr. Ajzen believed that behavioral intention shouldn’t be the exclusive determinant of our behaviors, especially that our control over behaviors isn’t complete. As a result, Dr. Ajzen improved the predictability of his theory by adding a new component, which he called the *Perceived Behavioral Control* [7], and that one in particular grew out of one of Dr. Bandura’s theories which is called *Self-Efficacy* [8].

Self-Efficacy is a theory developed by Dr. Bandura in the 1970s, and it became the center of his research on social cognitive [7]. Dr. Bandura distinguished several types of goal-oriented expectations: outcome expectancy and self-efficacy. Dr. Bandura defined outcome expectancy as our estimation that a certain behavior will lead to specific outcomes, and he defined self-efficacy as our belief in our own ability to accomplish something or succeed in a specific situation. The beliefs that some people hold regarding their power to make some change, strongly influence their actual power to face a challenge, as well as the choice they are most probably to make. Dr. Bandura advanced the view that goal-oriented expectations are the most important predictors and preconditions for behavioral change, which is the key to the initiation of coping behaviors [7,8].

The TPB posits that behavioral intention is influenced by three core components: the subjective norms regarding the behavior, the individual’s attitude toward the behavior, and perceived behavioral control [2,3]. Attitudes reflect the perceived benefits or drawbacks of performing the behavior; subjective norms capture a perceived social pressure to perform or abstain from the behavior; and perceived behavioral control denotes the perceived difficulty or ease of performing the behavior [3,4]. These components interact to predict the likelihood of engaging in a specific behavior [9–11].

Previous research has increasingly applied the Theory of Planned Behavior to disaster preparedness and other protective behaviors, generally demonstrating that the theory provides a useful framework for explaining preparedness intentions [5,9]. However, the relative importance of attitude, subjective norms, and perceived behavioral control has varied across different hazards, populations, and preparedness behaviors, suggesting that contextual factors influence the predictive strength of each construct [6,8]. These variations highlight the importance of evaluating the TPB within specific disaster contexts rather than assuming that findings are transferable across hazards. Hurricane evacuation planning involves unique social, logistical, and environmental challenges that may influence behavioral intentions differently from other preparedness actions. Examining the TPB in this context therefore provides an opportunity to strengthen both theoretical understanding and emergency management practice [7,10].

Attitudes can significantly impact individuals’ preparedness intentions and evacuation decisions. For instance, Wang et al. found that negative attitudes toward precautionary measures detract from residents’ willingness to protect against flooding in their community, thereby reducing preparedness levels [12]. Similarly, Brodar et al. highlighted that personal histories of disruption, such as experiences from prior storms, positively shaped people’ attitudes toward evacuation, leading them to express greater future evacuation intentions [13].

Similarly, subjective norms encompass the social influencers that shape individuals’ decisions in times of crisis. Identifying how peer expectations and community standards affect evacuation intentions is crucial. Armstrong et al. conducted research indicating that social validation and peer behaviors substantially influence individual perceptions of risk [14]. In the context of hurricanes, the behavior of surrounding individuals—whether they choose to evacuate or stay—can create a ripple effect, impacting collective decision-making processes. Furthermore, an integrative framework by Dicarlo and Berglund emphasized that utilization of social media platforms during disasters compels people to act on social cues from their network, thus impacting their evacuation decisions [15]. This underscores the role of community dynamics in shaping subjective norms that inform evacuation intentions.

On the other hand, perceived behavioral control refers to the extent to which individuals feel they have control over the difficulties associated with evacuating. Stewart elucidated how self-efficacy—an individual's belief in their ability to prepare for hurricanes—serves as a critical factor influencing evacuation likelihood [8]. Residents with prior experiences of evacuation reported a heightened sense of self-efficacy, which subsequently impacted their preparedness behaviors and intentions to evacuate in future events. Additionally, Kurata et al. demonstrated in their examination of flood-prone areas in the Philippines that factors such as prior knowledge of evacuation procedures and access to resources significantly enhance perceptions of control, thereby encouraging preparedness [16]. This relationship supports the TPB assertion that enhancing an individual’s perceived control can strengthen their behavioral intentions toward evacuation.

Understanding evacuation behavior through the lens of TPB is critical because evacuation remains one of the most effective strategies for reducing injury and loss of life during severe storms. Research has shown that non-evacuation is often not driven by ignorance of risk, but by behavioral and social factors such as social influence [17]. TPB provides a structured approach for examining these factors by linking beliefs and perceptions directly to behavioral intention. By identifying how attitudes toward evacuation, perceived social pressure, and perceived control over evacuation logistics influence intentions, emergency managers can better understand why individuals choose to evacuate or remain in place despite official warnings.

This behavioral understanding has important implications for the design of emergency management and evacuation planning. Emergency plans that focus solely on hazard communication may be insufficient if they fail to address the behavioral drivers of decision-making [17]. Integrating TPB-informed insights into emergency planning can support the development of targeted risk communication strategies, norm-based messaging, and interventions that reduce perceived barriers to evacuation. Expanding the application of the Theory of Planned Behavior to disaster contexts can therefore strengthen the behavioral foundation of emergency planning, resulting in more effective, people-centered strategies that enhance preparedness, improve evacuation compliance, and ultimately save lives during storms.

Taken together, the existing literature suggests that attitude, subjective norms, and perceived behavioral control may influence evacuation-planning intentions. However, empirical evidence applying the TPB specifically to hurricane-related evacuation planning remains limited. Therefore, this study examines whether these three TPB constructs predict intentions to prepare evacuation plans among residents of hurricane-prone areas. By evaluating the predictive utility of the TPB in this context, the study aims to determine whether the theory can provide a useful behavioral foundation for future emergency management interventions and risk communication strategies. The following section describes the study design, questionnaire, data-collection procedures, and statistical analyses used to evaluate these relationships.

## 3. Materials and methods

The main research question is: “Can the TPB predict people’s intentions of preparing evacuation plans in hurricane-prone areas?”, and to answer that question, a quantitative approach was utilized for the data collection in this research study, involving an online questionnaire that was filled by adult subjects (18 years and older) residing throughout the state of Florida, which is the most impacted state by storms in the US [17]. The choice was made to conduct the survey in digital online platform to get a higher number of responses.

### 3.1. Questionnaire design

The questionnaire items were adapted from previously published Theory of Planned Behavior instruments and modified to reflect the context of hurricane evacuation planning [5,6]. The wording of the items was refined to ensure relevance to evacuation preparedness while maintaining consistency with the conceptual definitions of attitude, subjective norms, and perceived behavioral control proposed by Ajzen [1]. Questions in the questionnaire were short/direct. The questionnaire had two main sections; the storm preparedness section, and the demographics section. Variables were measured through a set of questions in a Rising scales format (see Table 1). Survey Face Validity (SFV) was established by asking (5) researchers who understand the background of our research study to read through the questionnaire questions and evaluate whether the questions capture the topic we are investigating. Then, the survey was pilot tested on 11 subjects to make sure that the online questionnaire was clear, direct, and free from typos.

**Table 1:** Questions used in the survey to evaluate the TPB.

Section	Question	Response Scale	Scale Anchors
**Attitude**	During a hurricane, how necessary do you believe having an evacuation plan?	1–5	1 = Very unnecessary, 5 = Very necessary
	During a hurricane, how beneficial do you believe having an evacuation plan?	1–5	1 = Very unbeneficial, 5 = Very beneficial
	When it comes to saving lives and reducing physical harm, how effective is an evacuation plan?	1–5	1 = Very ineffective, 5 = Very effective
**Subjective Norms**	During the hurricane season, how many people around you do you believe have evacuation plans?	1–5	1 = No one, 5 = Everyone
	How many of your friends and family members think you should have an evacuation plan during the hurricane season?	1–5	1 = No one, 5 = Everyone
	During the hurricane season, how likely is it that you will be motivated to have an evacuation plan if people around you have one?	1–5	1 = Not at all, 5 = Very Likely
**Perceived Behavioral Control**	How likely is it that you will be able to prepare an evacuation plan?	1–5	1 = Not at all, 5 = Very Likely
	How much time do you believe it would take you to prepare an evacuation plan?	1–5	1 = Little, 5 = A lot
	How much effort do you believe it would take you to prepare an evacuation plan?	1–5	1 = Little, 5 = A lot
	How overwhelmed would you be if you were to prepare an evacuation plan?	1–5	1 = Little, 5 = A lot
**Intention**	During the next hurricane season, how likely is it that you will prepare an evacuation plan?	0–10	0 = Not at all, 10 = Very Likely

Note: For the perceived behavioral control scale, the negatively oriented items assessing the time, effort, and feeling of being overwhelmed associated with preparing an evacuation plan were reverse-coded. Reverse-coded scores were calculated as 6 minus the original score. The positively oriented item assessing participants’ perceived ability to prepare an evacuation plan was not reverse-coded.

In the survey, the participants were provided with the following definition for more clarity: “*Preparing an evacuation plan. Having an evacuation plan means you need to: 1) become familiar with the evacuation routes and locations of hurricane shelters near you, 2) know when you are supposed to evacuate your dwelling, 3) know what means of transportation you might use to reach any of the nearby safe shelters, 4) communicate your plan with your roommates, family, and friends, and 5) practice going to a nearby hurricane shelter*.”

The independent variables were the three TPB constructs. Attitude was operationalized as the mean of three 1–5 items assessing the perceived necessity, benefit, and effectiveness of having an evacuation plan, with higher scores indicating a more favorable attitude. Subjective norms were operationalized as the mean of three 1–5 items assessing perceived evacuation-planning behavior among people in the respondent’s social environment, perceived expectations of family and friends, and motivation to prepare when others have an evacuation plan. Higher scores indicated stronger perceived social influence supporting evacuation planning. Perceived behavioral control was operationalized as the mean of four 1–5 items assessing perceived ability, anticipated time, anticipated effort, and anticipated feelings of being overwhelmed when preparing an evacuation plan. The time, effort, and feeling-overwhelmed items were reverse-coded using the transformation 6−original score, so that higher scores consistently indicated greater perceived behavioral control and lower perceived barriers.

The perceived behavioral control items were designed to capture both perceived capability and perceived ease or difficulty associated with preparing an evacuation plan. The item assessing participants’ perceived ability to prepare a plan measured capability, whereas the items assessing anticipated time, effort, and feelings of being overwhelmed measured perceived task demands and barriers. Respondents who expected the task to require little time and effort, would not make them feel overwhelmed, and was within their ability to accomplish were considered to perceive the task as relatively easy. Conversely, expecting substantial time or effort or feeling overwhelmed indicated greater perceived difficulty.

The demographic variables were selected because previous disaster preparedness research has shown that age, gender, race/ethnicity, and prior exposure to hurricane-prone environments may influence preparedness behaviors and evacuation intentions [4–12]. These variables were therefore included to characterize the study sample and provide context for interpreting the findings.

### 3.2. Data collection and analysis

The study employed a convenience sampling approach, whereby the survey was made available online to subjects (18 years or older) in Florida using several social media platforms (Facebook and LinkedIn) and with help from Florida Climate Institute, who agreed to help in the data collection. Identical survey links were used across so the number of respondents generated by each individual platform could not be determined. Moreover, the questionnaire was administered in English only. Our survey recruitment was from 8/29/2022–12/12/2022. As mentioned before, each behavioral construct was measured through a set of questions in a Likert or Rising scales format (see Table 1). The answers were translated into percentages, for example, Very Unnecessary, (0.0%), Unnecessary (25%), Neither Necessary nor Unnecessary (50%), Necessary (75%), and Very Necessary (100%). The average (%) of the questions’ set for each variable was assigned as the variable’s final score, which we later used in the analysis. The reliability (or internal consistency) of each multi-item scale was assessed using Cronbach's alpha, with a minimum acceptable value of 0.70 indicating satisfactory reliability.

Different statistical approaches (through DataTab & SPSS software package) were used, including Spearman correlations and binary logistic regression, so that we uncover any patterns and quantify the behavioral constructs based on generating numerical data to quantify behaviors and attitudes [18,19]. For the logistic regression, preparedness intentions were categorized as the following: scores below 40% (or 0.4) equals “No” or “Will Not Prepare,” scores between 40% and 60% (0.4–0.6) equals “Indecisive,” and scores above 60% (or 0.6) equals “Yes” or “Will Prepare.” The “No” or “Will Not Prepare” were assigned a value of 0 in the logistic regression, while “Yes” or “Will Prepare” were assigned a value of 1. The values that came out as “Indecisive” were not included the logistic regression.

This approach established a buffer around the midpoint and minimized the potential misclassification that could occur when scores are dichotomized at 0.50. A simple 50% cutoff could classify participants with scores close to the midpoint—such as 0.49 and 0.51—into opposite outcome groups, despite their nearly identical preparedness intentions, potentially affecting the stability and interpretation of the regression results. This approach has been recognized and published by the Natural Hazards Center [19], as well as other disaster studies that are published with leading academic journals in this field [18].

The dependent variable in this study was the intention to prepare an evacuation plan during the next hurricane season. The independent variables were the three Theory of Planned Behavior constructs: attitude toward evacuation planning, subjective norms, and perceived behavioral control. For the Spearman correlation analysis, associations between each TPB construct and preparedness intention were examined. For the logistic regression analysis, preparedness intention served as the dependent variable, while attitude, subjective norms, and perceived behavioral control were entered simultaneously as predictor variables to evaluate their relative contributions to evacuation planning intentions.

### 3.3. Ethics statement

We received ethical approval for our research from the IRB at University of Florida (reference number IRB202200544). Informed consent was obtained from all survey participants, in the knowledge that we did not collect identifiable data to protect the confidentiality of the study subjects. Artificial intelligence tools were used to assist with organizing and formatting the manuscript; all substantive content, analysis, and interpretations were developed by the author.

The methods described above enabled us to operationalize the three TPB constructs and assess their relationships with intention to prepare an evacuation plan. Spearman correlation analysis was used to examine the associations among the constructs, while binary logistic regression was used to evaluate their relative contributions to evacuation-planning intention. The results of these analyses are presented in the following section.

## 4. Results

A total of 979 participants started the survey, of whom 816 completed it. The survey sample was predominantly female (62.37%), and the majority identified as White (53.22%). Around 50% of the sample was 24 years or younger. The majority lived in hurricane-prone territories for over 10 years (60.81%). See Table 2 for subjects’ demographics.

**Table 2:** Sample demographics (N = 816).

Demographics	Frequency	
	n	%
Age		
19 or less	238	30.51%
20 - 24	170	21.79%
25 - 29	106	13.59%
30 - 34	65	8.33%
35 - 39	46	5.90%
40 - 44	36	4.62%
45 - 49	26	3.33%
50 - 54	24	3.08%
55 - 59	22	2.82%
60 - 64	22	2.82%
65+	25	3.21%
Total	780	100%
Gender Identity		
Female	474	62.37%
Male	269	35.39%
Non-binary	17	2.24%
Total	760	100%
Race/Ethnicity		
White	405	53.22%
Asian	77	10.12%
South Asian	36	4.73%
Latino	76	9.99%
Black	75	9.86%
Mixed Race	92	12.09%
Total	761	100%
Living in Hurricane Prone Area		
Less than 2 years	134	17.05%
2 - 5 years	96	12.21%
6 - 10 years	78	9.92%
More than 10 years	478	60.81%
Total	786	100%

*Note: Demographic totals differ from the overall sample size (N = 816) because some participants chose not to answer one or more demographic questions. Percentages are therefore based on the number of valid responses for each demographic variable.*

Internal consistency was tested for all multi-item constructs, with Cronbach’s alpha values of 0.91 for attitude, 0.85 for subjective norms, and 0.86 for perceived behavioral control (see Table 3). Additionally, we used Spearman’s correlation to test the relationship between each of behavioral constructs and the intention of preparing an evacuation plan (see Table 4). Attitude was moderately and positively correlated with subjective norms (r = 0.47, p < .001) and perceived behavioral control (r = 0.48, p < .001), indicating that more favorable attitudes toward evacuation planning were associated with stronger social influences and higher preparedness intentions. Subjective norms showed a strong positive relationship with intention (r = 0.55, p < .001), suggesting that perceived social expectations play a substantial role in motivating evacuation planning.

**Table 3:** Internal consistency reliability (N = 816).

Variable	Cronbach's Alpha
Attitude	0.91
Subjective Norms	0.85
Perceived Behavioral Control	0.86

**Table 4:** Correlation and significance (Preparing an evacuation plan).

Variable		1. Attitude	2. Subjective Norms	3. Perceived Behavioral Control	4. Intention
1. Attitude	Correlation	1	0.47	−0.02	0.48
	p	<.001	<.001	0.481	<.001
2. Subjective Norms	Correlation	0.47	1	0.09	0.55
	p	<.001	<.001	0.011	<.001
3. Perceived Behavioral Control	Correlation	−0.02	0.09	1	0.31
	p	0.481	0.011	<.001	<.001
4. Intention	Correlation	0.48	0.55	0.31	1
	p	<.001	<.001	<.001	<.001

-(r)-value: Very weak (0,0 < 0,1), Weak (0,1 < 0,3), Medium (0,3 < 0,5), Strong (0,5 < 0,7), Very strong (0,7 < 1)

-Insignificant correlation (p-value > 0.01)

Perceived behavioral control demonstrated a moderate positive correlation with intention (r = 0.31, p < .001), indicating that individuals who felt more capable of preparing an evacuation plan were more likely to intend to do so. In contrast, perceived behavioral control was not significantly correlated with attitude (r = −0.02, p = .481) and showed only a weak relationship with subjective norms (r = 0.09, p = .011), suggesting that perceived ability operates largely independently of evaluative beliefs and social pressures. Overall, the findings highlight subjective norms and attitude as the strongest correlates of intention to prepare an evacuation plan.

Table 5 presents the results of the logistic regression analysis examining predictors for intending to prepare an evacuation plan. As mentioned before, when it comes to preparedness intentions and logistic regression, the “No” or “Will Not Prepare” were assigned a value of 0, and those were 303 responses in total, while “Yes” or “Will Prepare” were assigned a value of 1, and those were 221 responses in total. The values that came out as “Indecisive” were not included in the logistic regression. Accordingly, the analytical N in the logistic regression analysis was (N = 303 + 221 = 524).

**Table 5:** Logistic regression (Intention to prepare an evacuation plan).

Model	Mean	SD	Coefficient B	Standard error	z	p	Odds Ratio	95% conf. interval
Perceived Behavioral Control	60%	22%	4.17	0.63	6.62	<.001	64.74	18.86 - 222.27
Attitude	72%	23%	4.55	0.71	6.41	<.001	94.2	23.22 - 382.19
Subjective Norms	54%	20%	5.98	0.78	7.67	<.001	396.31	85.15 - 1844.47
Constant	–	–	−9.91	0.94	10.58	<.001	–	–
								
Chi2	df		p	−2 Log-Likelihood			Adjusted R^2^ (Nagelkerke R^2^)	
322.24	4		<.001	391.29			0.62	

-Significant (p-value < 0.01)

-R^2^ Value: Very weak (0,0 < 0,1), Weak (0,1 < 0,3), Medium (0,3 < 0,5), Strong (0,5 < 0,7), Very strong (0,7 < 1)

-The analytical N in the logistic regression analysis was 524.

The overall model was statistically significant (p < .001) and demonstrated strong explanatory power, with a Nagelkerke R^2^ of 0.62, indicating a very strong model fit. All three predictors—perceived behavioral control, attitude, and subjective norms—were statistically significant (p < .001). Subjective norms emerged as the strongest predictor of evacuation plan preparation (OR = 396.31), suggesting that individuals who perceived stronger social expectations were substantially more likely to intend to prepare an evacuation plan.

Attitude also showed a strong positive effect (OR = 94.20), indicating that more favorable views toward evacuation planning greatly increased the likelihood of preparedness. Perceived behavioral control was likewise a significant predictor (OR = 64.74), demonstrating that greater confidence in one’s ability to prepare an evacuation plan substantially increased the odds of intending to prepare an evacuation plan. The logistic regression is in agreement with the Spearman’s correlation (See Table 4).

Demographic characteristics were collected to describe the composition of the study sample and provide context for interpreting the findings. Although age, gender, race/ethnicity, and prior hurricane-related experience may influence preparedness intentions and behaviors, the TPB treats these characteristics as background factors whose influence is expected to operate indirectly through the constructs of attitude, subjective norms, and perceived behavioral control. Accordingly, this study focused on evaluating whether the three TPB constructs predicted evacuation-planning intention rather than estimating the independent effects of demographic characteristics. Demographic variables were therefore not included as covariates in the primary model. Including them would estimate the TPB associations conditional on these background factors and could account for part of the pathway through which demographic characteristics shape TPB beliefs.

Overall, the findings indicate that attitude, subjective norms, and perceived behavioral control were positively associated with respondents’ intention to prepare an evacuation plan. Subjective norms showed the strongest association, followed by attitude and perceived behavioral control. The following section interprets these findings in relation to the TPB literature and considers their implications for emergency-management practice.

## 5. Discussion

The findings from this research study contribute valuable insights into the application of the TPB in the context of disaster preparedness, specifically in relation to evacuation intentions during hurricanes. As highlighted in the literature review, the TPB comprises three key components—attitude, subjective norms, and perceived behavioral control—that interact to shape behavioral intentions [2–5]. This study confirms the TPB's robustness and applicability in predicting how individuals prepare for evacuation plans in hurricane-prone areas and suggests important implications for emergency management practices.

The data analysis revealed strong predictive relationships between the TPB components and individuals’ intentions to prepare evacuation plans. Specifically, subjective norms were found to be the strongest predictor of evacuation preparedness intention, suggesting that social influences and community expectations significantly drive individuals to take proactive measures. These findings are consistent with those of Huang, who emphasized that social pressures play a critical role in evacuation decision-making [20]. In real-world emergencies, individuals often look to their peers and the people around them as a social guide [21–24]. This bandwagon effect bolsters the argument that community dynamics should be interpreted and managed within the emergency preparedness framework.

Moreover, the correlation between attitudes toward evacuation planning and positive intentions to prepare was also significant. In our study, individuals who held favorable attitudes about the benefits of having an evacuation plan were more likely to have stronger intentions to prepare. Attitudes can also influence others through interpersonal communication [17]. As individuals discuss preparedness with family, friends, or members of their community, they are exposed to and influenced by one another's perspectives. Accordingly, individuals with strong positive attitudes toward evacuation planning may help shape the attitudes of those around them, increasing the likelihood that others will also adopt preparedness behaviors. This reinforces the idea that public perception can be shape-shifting based on individual experiences, fears, or stories shared within communities [23–26]. As indicated by Cuite perceptions of storm severity affect preparedness behaviors, suggesting that emergency communication strategies should not just inform but actively engage communities to foster positive attitudes toward preparedness [27].

Perceived behavioral control was another significant predictor of individuals’ intentions to prepare, echoing findings in the literature that highlight self-efficacy's role in influencing preparedness behaviors [28,29]. When individuals believe they can successfully create an evacuation plan, their commitment toward executing those plans intensifies. This introduces the notion that efforts to increase self-efficacy—by providing resources, education about evacuation routes, or simulation practices—can directly influence evacuation preparedness intentions. Research by Yang supports this assertion [30], indicating that enhancing knowledge and skills through practical engagement in disaster preparedness training programs results in higher self-efficacy and, consequently, more robust preparedness behaviors.

The prominence of subjective norms in this study may reflect a distinctive characteristic of hurricane preparedness compared with many routine health or environmental behaviors where personal attitudes or perceived control often play a larger role. Hurricane evacuation decisions are rarely made in isolation. Residents continuously observe the actions, expectations, and experiences of family members, neighbors, coworkers, and local communities before deciding whether and how to prepare. In Florida, where hurricanes are recurrent events, preparedness behaviors may become socially reinforced through repeated community experiences rather than being driven solely by individual risk assessments. This helps explain why perceived social expectations emerged as a stronger predictor of evacuation planning intentions than either personal attitudes or perceived behavioral control.

Even though individuals may recognize the benefits of having an evacuation plan and believe they are capable of creating one, these beliefs may not translate into action unless they are supported by visible social norms and collective preparedness practices. The comparatively less strong association observed for perceived behavioral control and attitude may also suggest that many residents already possess sufficient knowledge or resources to prepare, reducing variability in perceived capability across the sample. Under these circumstances, the deciding factor becomes whether preparedness is viewed as a socially expected behavior. For emergency managers, these findings indicate that preparedness campaigns should leverage community leaders, neighborhood organizations, and peer-to-peer messaging to establish evacuation planning as a visible and widely accepted social norm rather than relying exclusively on informational campaigns.

The implications for emergency management policy and practice are profound. With evidence indicating that subjective norms are pivotal in shaping evacuation intentions, emergency management authorities should consider strategies that leverage these community influences [21,26]. Initiatives might include community-focused campaigns that highlight stories of neighbors or local community leaders who have successfully prepared for storms. Such narratives can significantly enhance the acceptability and relevance of evacuation planning, nurturing social norms that favor preparedness [31].

Effective communication strategies underpin successful evacuation behaviors. Factors such as using clear risk messages and leveraging social media can enhance public comprehension and perception, subsequently influencing evacuation decisions. Kato explored the impact of information strategies in promoting disaster management measures and highlighted the critical role of informational outreach in enhancing preparedness and evacuation behavior among residents [32]. Moreover, the work of Dang elaborates on how the credibility of information disseminated influences individual perceptions regarding risks, stressing that ensuring reliable messaging can be paramount in fostering resilience during crisis situations [33].

Furthermore, addressing barriers associated with perceived behavioral control should also be a priority. Emergency managers need to devise clear, accessible public education campaigns that demystify the evacuation planning process [34]. Breakdowns in complex information regarding evacuation routes, available shelters, and modes of transport could reduce perceived barriers and enhance individuals’ ability to prepare adequately. This is critical, especially for vulnerable populations with limited mobility or resources, as indicated in previous research about disaster preparedness disparities [35,36].

These findings also have important equity implications. Individuals with limited access to transportation, reduced mobility, financial constraints, or rental housing may experience lower perceived behavioral control because they face greater logistical barriers to evacuation planning. For these populations, interventions should extend beyond information dissemination to include practical resources such as transportation assistance, accessible evacuation route planning, shelter accessibility information, and simplified preparedness checklists. By reducing structural barriers while simultaneously increasing individuals’ confidence in their ability to prepare, emergency management agencies can strengthen perceived behavioral control and promote more equitable preparedness outcomes across diverse communities.

Additionally, implementing training programs focused on building self-efficacy in disaster risk management could be an effective intervention [21,37,38]. By simulating evacuation scenarios and providing hands-on tools to develop plans, emergency management agencies can demonstrate viable pathways for residents to mitigate storm-related risks [26,39–41]. Recent studies suggest that exposure to similar training increases preparedness behavior through elevated self-efficacy levels among participants [18,29,40–42].

While the study provides novel insights, it also presents several limitations. This reliance on self-reported data introduces some potential biases, as individuals may overestimate their preparedness intentions. This study relied on self-reported data, which may be subject to social desirability and recall biases, potentially leading some participants to overestimate their preparedness intentions. Several measures were implemented to minimize these biases, including anonymous participation, the absence of personally identifiable information, voluntary participation with informed consent, and the use of a pilot-tested questionnaire consisting of short and clearly worded questions. Nevertheless, self-reported intentions may not fully reflect actual preparedness behaviors and should therefore be interpreted with appropriate caution.

Another limitation relates to the composition of the study sample. Compared with Florida, this study included a higher proportion of women (62.37% vs. 51.3%) and a lower proportion of men (35.39% vs. 48.7%). In addition, the study had higher proportions of White participants (53.22% vs. 49.9%), higher proportions of Asian participants (14.8% vs. 3.5%), lower proportions of Black participants (9.86% vs. 16.3%), lower proportions of Latino participants (9.99% vs. 29.2%), and higher proportions of participants identifying as mixed race (12.09% vs. 4.0%) [43].

The sample’s gender composition differs from Florida’s population, and census data do not provide a comparable category for non-binary participants as our study. Racial and ethnic composition also differs from Florida’s, including Black, Latino, Asian, South Asian, and mixed-race representation. These differences may mean results do not fully reflect all Floridians’ experiences or perspectives. Census categories also do not align perfectly with the study’s categories, which limits direct comparisons. Therefore, the findings may be applicable to populations with characteristics similar to those of the participants; broader conclusions require replication in more diverse samples. In addition, household income was not collected, preventing assessment of how socioeconomic differences may affect evacuation planning intentions. Consequently, the observed relationships should be interpreted with appropriate caution. Future studies should seek more demographically representative samples across age, gender, and other population characteristics to determine whether the observed behavioral patterns remain consistent in broader populations.

Our logistic regression models analyzed respondents who expressed a position (“Will Prepare” or “Will not Prepare”), and those were 524 responses in total. However, indecisive responses (n = 292) were outside the scope of our model. This focus enabled examination of factors associated with defined response categories and points to a valuable direction for future research: understanding indecision. Future studies could model indecisive responses as a distinct outcome and explore how uncertainty relates to the behavioral constructs in the model. Such work would complement our findings and broaden understanding of respondents’ broader perspectives.

Moreover, future research could extend the TPB framework by incorporating additional factors such as emotional responses and risk perception dynamics to provide a comprehensive understanding of evacuation behaviors in disaster scenarios. Incorporating qualitative approaches could also enrich findings, capturing in-depth narratives from diverse populations about their experiences and perceptions regarding storm evacuations. Future qualitative research, including interviews, focus groups, or open-ended survey responses, could provide deeper insight into how individuals reason through evacuation planning decisions. Such approaches would help explain the behavioral mechanisms underlying the observed statistical associations, particularly within communities where strong social relationships influence preparedness decisions.

This qualitative data could help emergency management officials better tailor communication strategies to address specific community concerns and challenges. Moreover, exploring demographic variations within the framework of TPB might highlight pertinent differences in preparedness behavior among different social groups, thereby enhancing the design of more inclusive and effective intervention strategies. Furthermore, demographic variables were not included as covariates in the regression model because the primary objective of this study was to evaluate the predictive utility of the core Theory of Planned Behavior constructs. Accordingly, the analysis focused on examining the independent contributions of attitude, subjective norms, and perceived behavioral control to evacuation planning intentions. Future studies may want to incorporate demographic covariates to determine whether these relationships remain consistent across different population subgroups. Finally, the predictive performance of the logistic regression model was evaluated only within the study sample. Future research should assess the robustness and generalizability of the model using independent datasets or cross-validation techniques to confirm its predictive performance across different populations and disaster contexts.

In summary, the findings support the usefulness of the TPB for understanding evacuation-planning intentions. However, because the study measured intention rather than actual preparedness or evacuation behavior, the findings should be interpreted accordingly. The conclusion that follows summarizes the study’s principal contributions and implications.

## 6. Conclusion

This study provides strong empirical support for the application of the TPB in explaining and predicting evacuation planning intentions in hurricane-prone areas. By examining the roles of subjective norms, attitude, and perceived behavioral control, the findings demonstrate that evacuation preparedness is not solely a function of risk awareness, but is deeply shaped by social influence, personal evaluations, and perceived capacity to act. The results reinforce TPB as a robust and transferable framework for understanding disaster-related decision-making under conditions of uncertainty and stress. Across all statistical analyses, subjective norms emerged as the most influential predictor of evacuation planning intention. This highlights the powerful role of social context in shaping preparedness behaviors, suggesting that individuals are strongly motivated by the expectations and actions of those around them.

Attitude toward evacuation planning also showed a significant and positive effect, indicating that individuals who perceive evacuation plans as beneficial, necessary, and effective are more likely to intend to prepare. Perceived behavioral control further contributed to intention, underscoring the importance of self-efficacy and the perceived ability to overcome logistical and cognitive barriers associated with evacuation planning. These findings have important implications for emergency management policy and practice. Traditional preparedness strategies that focus primarily on disseminating hazard information may be insufficient if they fail to address the behavioral drivers identified in this study. Emergency communication efforts should leverage social norms by highlighting community preparedness, peer behavior, and local leadership engagement. Additionally, interventions aimed at improving attitudes toward evacuation planning—such as emphasizing its life-saving benefits—can further strengthen preparedness intentions. Enhancing perceived behavioral control through clear guidance, accessible planning tools, and practical training opportunities may be particularly effective for increasing preparedness among populations facing resource or mobility constraints.

## Supporting information

S1 File Dataset (N = 816). Survey responses.
(XLSX)
